# Cost-effective method for semi-quantitative analysis of soluble endoglin in biological samples after anti-endoglin monoclonal antibody treatment

**DOI:** 10.1038/s41598-025-21972-w

**Published:** 2025-10-30

**Authors:** Samira Eissazadeh, Jana Urbankova Rathouska, Ivana Nemeckova, Petra Fikrova, Katarina Tripska, Martina Vasinova, Martina Kolackova, Petr Nachtigal

**Affiliations:** 1 Department of Biological and Medical Sciences, Faculty of Pharmacy in Hradec Kralove, Charles University, Heyrovskeho 1203, 500 05 Hradec Kralove, Czech Republic; 2 Department of Clinical Immunology and Allergology, Faculty of Medicine in Hradec Kralove, Charles University, Hradec Kralove, Czech Republic

**Keywords:** Soluble endoglin, Cost-effective analysis, Anti-endoglin monoclonal antibody, Biochemistry, Biological techniques, Biomarkers, Biotechnology

## Abstract

Soluble endoglin (sENG) is an important biomarker of several cardiometabolic and vascular disorders. The accurate measurement of biomarkers that are simultaneously targeted by therapeutic monoclonal antibodies (mAbs) in preclinical models is a significant challenge. Traditional enzyme-linked immunosorbent assays (ELISAs) often fail due to epitope blocking, while advanced techniques like mass spectrometry are more expensive and require specialized expertise. To address these limitations, we developed a cost-effective, specific Western blot-based method for evaluating sENG in mouse plasma in a metabolic dysfunction-associated steatohepatitis (MASH) rescue model. Thus, from a methodological perspective, we significantly modified and extended the sENG detection method introduced in our previous work, focusing on developing a cost-effective approach for semi-quantitative analysis. For these purposes, we used a mouse MASH model treated with the anti-endoglin (ENG) mAb M1043. Plasma samples were concentrated, and proteins were separated by sodium dodecyl sulphate polyacrylamide gel electrophoresis (SDS-PAGE) and probed with an anti-ENG antibody. We verified the method using human hepatic sinusoidal endothelial cells (HHSECs) culture media treated with the therapeutic human anti-ENG monoclonal antibody, TRC105. This Western blot-based approach avoids interference from therapeutic mAbs, delivering reliable and reproducible results. This method overcomes the limitations of ELISAs and mass spectrometer, providing a practical solution for semi-quantitative analysis of biomarkers in both preclinical and clinical research. Its versatility makes it applicable to various soluble protein biomarkers across diseases. As therapeutic mAbs become more widely used, this simple, cost-effective method facilitates mechanistic insights and accelerates targeted therapy development in research.

**Supplementary Information:**

The online version contains supplementary material available at 10.1038/s41598-025-21972-w.

## Introduction

Soluble endoglin (sENG), a circulating fragment of the transforming growth factor beta (TGF-β) co-receptor CD105/endoglin (ENG), profoundly influences angiogenesis, inflammation, and fibrosis, establishing it as a vital biomarker for metabolic dysfunction-associated steatohepatitis (MASH), cancer, preeclampsia, and vascular disorders^1–7^. Elevated sENG levels robustly correlate with disease severity and therapeutic response in preeclampsia^8^, and MASH and related conditions, necessitating comparative analysis in biological systems in preclinical models evaluating anti-ENG monoclonal antibodies (mAbs), such as M1043 and TRC105^7,9,10^. These mAbs, by modulating angiogenic, inflammatory, and fibrotic signalling pathways, hold therapeutic promise to mitigate disease progression, yet their evaluation depends on sENG measurement.

The uncertainty in quantifying biomarkers in the presence of therapeutic monoclonal antibodies complicates preclinical research, requiring innovative methods to address these challenges. Enzyme-linked immunosorbent assays (ELISAs) have been a keystone for sENG quantification due to their high sensitivity and throughput^5,11–17^. However, in models treated with mAbs, immunoassays are often compromised by epitope blocking between detection antibodies and therapeutic mAbs, which target the same or steric hindrance epitope, leading to unreliable data and hindering mechanistic understanding^18–20^. Indeed, Liu et al. showed that sENG cannot be detected in patients treated with an anti-ENG monoclonal antibody (TRC105) using the R&D Quantikine ELISA kit (DNDG00), but it can be detected with the Aushon CD105 Searchlight kit (multiplex ELISA), which is, however, no longer available^21^. Alternative approaches, such as liquid chromatography-tandem mass spectrometry (LC–MS/MS), offer superior specificity but require expensive equipment and specialized expertise, limiting their accessibility^22^. Similarly, proximity extension assays provide high sensitivity but are constrained by high costs and limited applicability^23,24^. These challenges underscore the need for an affordable, effective method to improve the accuracy of sENG quantification in mAb-treated models.

Western blot analysis provides a robust and accessible solution to these challenges. By resolving proteins based on molecular weight, this method avoids cross-reactivity, ensuring specificity in complex plasma samples. Western blot requires only standard laboratory equipment, offering a cost-effective alternative to LC–MS/MS and proximity assays^25,26^. This study demonstrates a straightforward and specific Western blot-based protocol for semi-quantifying sENG in mouse plasma from a MASH animal model, utilizing C57BL/6 J mice treated with mouse anti-ENG antibody M1043. To verify its reliability in in vitro conditions, the protocol was also applied to human hepatic sinusoidal endothelial cells (HHSECs) culture media treated with human anti-ENG antibody TRC105. This approach provides reproducible, interference-free results, overcoming the limitations of other techniques.

sENG is commonly measured using commercial ELISA kits. However, this approach has limitations when studying therapeutic interventions with mAbs, as Ab binding can mask ELISA epitopes and prevent accurate detection. In addition, ELISA cannot distinguish between free and Ab-bound ENG, complicating the interpretation of treatment outcomes. Although LC–MS/MS provides sensitive protein quantification, it requires specialized instrumentation, complex sample preparation, and remains inaccessible to many laboratories. To overcome these challenges, we propose here an optimized Western blot-based protocol that allows reliable and reproducible detection of sENG following mAb treatment, addressing a critical gap in available methods.

By shedding light on the pitfalls of conventional methods, this Western blot-based protocol paves the way for the semi-quantitative evaluation of biomarkers in mAb-treated models. Its simplicity and affordability provide access to reliable data, enabling researchers to explore various soluble protein biomarkers across diverse disease contexts. This method not only enhances the reliability of preclinical and clinical investigations but also fosters mechanistic insights that may catalyze the development of targeted therapies, lighting a path toward transformative clinical advancements.

In this study, we provide updated and modified methodological details about sENG analysis when compared to our previous paper^7^.

## Methods and materials

### Study design: in vivo and in vitro approaches

The study was designed to investigate the effects of dietary interventions and therapeutic treatments on MASH using both in vivo and in vitro approaches. This includes the animal housing protocol, MASH diet, and treatment with M1043, all based on methods described in Eissazadeh et al., 2025^7^, which demonstrated its reliability and reproducibility in modelling MASH pathophysiology. By reusing this well-established methodology, we adhered to the principles of the 3Rs (Replacement, Reduction, and Refinement), minimizing the use of additional animals while ensuring consistency and comparability with prior findings.

#### Animal study design

The animal study included three experimental groups, each consisting of ten-week-old male C57BL/6 J mice (Velaz, Prague, Czech Republic). The mice were housed under controlled environmental conditions (22 °C ± 1 °C, 12-h light/dark cycle, constant humidity) with free access to water and rodent diets. After a two-week acclimation period, eighteen mice were randomly divided into three groups (n = 6 per group).

The first group, referred to as the Control Group, was fed a standard chow diet (1314–10 mm pellets, Altromin, Lage, NRW, Germany) for 8 weeks. This group served as a baseline reference to represent normal physiological conditions in the absence of dietary or therapeutic manipulation. The second group, termed the MASH-Induced Group, was subjected to a choline-deficient, high-fat diet (CDAA-HFD; A06071302, Research Diets, New Brunswick, NJ, USA) to induce MASH. The third group, designated the Rescue Group, was also fed the CDAA-HFD to induce MASH but was treated with intraperitoneal injections of M1043 (10 mg/kg; TRACON Pharmaceuticals Inc.) twice weekly for 4 weeks after MASH induction (as previously described in Eissazadeh et al., 2025^7^. This group was designed to evaluate the therapeutic efficacy of M1043 in mitigating disease progression and alleviating pathological changes associated with MASH. By comparing the outcomes across the three groups, the study aimed to elucidate the mechanistic basis of MASH pathophysiology and assess the potential of M1043 as a targeted therapeutic intervention.

At the end of the 8-week study period, all mice were euthanized via anesthetic overdose (xylazine 10 mg/kg and ketamine 100 mg/kg, i.p.), and blood was collected from the *inferior vena cava*. Blood samples were processed in EDTA-coated tubes and centrifuged at 2000 × g for 10 min at 4 °C to separate plasma. Plasma aliquots were stored at − 80 °C until further analysis. The sample size (n = 6 per group) was determined using G*Power software based on a non-parametric Mann–Whitney test (α = 0.05) (based on power analysis similar to that reported in our previous study^7^, ensuring sufficient statistical power to detect meaningful differences between groups. All procedures adhered to the principles of the 3Rs and the Five Freedoms for animal welfare^27^. The study was approved by the Charles University Ethical Committee, Faculty of Pharmacy in Hradec Kralove (Project MSMT-5793/2021–2, approval date: 4 May 2021), and complied with Czech Law No. 246/1992 Sb. The protocol was registered and approved in the International Register of Preclinical Trial Protocols (PCTE0000526). This rigorous ethical framework ensured that the study minimized animal use while maximizing the reliability and reproducibility of the findings. All experimental procedures were conducted in full accordance with relevant institutional and national guidelines and regulations. In addition, all animal experiments were reported in compliance with the ARRIVE guidelines (https://arriveguidelines.org) to ensure transparency and reproducibility.

#### In vitro study design

To complement the in vivo findings, an in vitro study was conducted using HHSECs. This experimental setup was adapted from the previously published study^7^, which successfully modelled HHSECs inflammation and dysfunction in the context of MASH. To mimic inflammatory conditions observed in MASH, HHSECs were treated with ox-LDL (L34357, ThermoFisher Scientific, Waltham, MA, USA) at a concentration of 50 μg/mL for 48 h. HHSECs were pretreated with the anti-human ENG monoclonal antibody TRC105 (300 μg/mL; TRACON Pharmaceuticals Inc.) for 3 h before being exposed to ox-LDL, as a part of a rescue experiment. Control cells were handled under identical conditions, including media replacement, but were not subjected to any treatment.

This in vitro approach was selected not only for its proven ability to replicate key aspects of HHSECs dysfunction observed in vivo, but also because it provides a platform for expanding the investigation in vitro as well.

### Western blot-based analysis

This protocol was specifically optimized for situations in which ELISA detection is compromised due to mAb interference and where LC–MS/MS is impractical due to instrumentation requirements. Key modifications, including pre-concentration of plasma and culture media, refined lysis, blocking, and antibody incubation conditions, ensure accurate detection of sENG under these experimental conditions while maintaining reproducibility across murine and human samples.

#### Plasma sample concentration

To ensure sufficient protein content for accurate detection, both plasma and culture media samples were concentrated before Western blot analysis. For this purpose, 100 μL aliquots of each sample were transferred to 1.5 mL centrifuge tubes and placed in a Vacufuge Concentrator 5301 (Eppendorf, Hamburg, Germany). The samples were first concentrated at room temperature for 30 min, followed by an additional round at 30 °C. Specifically, mouse plasma samples were concentrated for 30 min at 30 °C, while culture media samples required an extended concentration period of 50 min at 30 °C to achieve optimal protein content. Protein concentrations were subsequently quantified using a Pierce™ BCA Protein Assay Kit (ThermoFisher Scientific, Waltham, MA, USA) to ensure accuracy and consistency across all samples.

#### Western blot analysis

Concentrated plasma samples containing 30 μg of protein per sample were diluted in Laemmli sample buffer (Sigma-Aldrich, MA, USA), boiled at 95 °C for 5 min, and separated by SDS-PAGE on 6.25% polyacrylamide gels (Bio-Rad Laboratories, Hercules, CA, USA), which were selected based on the molecular weight of measured proteins. Then, proteins were transferred to nitrocellulose membranes (Bio-Rad Laboratories, Hercules, CA, USA) using a wet transfer system at 140 V and 300 mA for 1 h and 45 min. The membranes were then blocked with 5% non-fat dry milk (Bio-Rad Laboratories, Hercules, CA, USA) in Tris-Buffered Saline with 0.1% Tween 20 (TBS-T; MilliporeSigma, Burlington, MA, USA) for 1 h and 15 min at room temperature to reduce nonspecific binding.

For protein detection, the membranes were incubated overnight at 4 °C with primary antibodies targeting specific proteins. Mouse sENG was detected using anti-CD105 (ab221675, Abcam, Cambridge, UK) at an optimized concentration of 1:800, human sENG was detected using a primary antibody from Abcam (ab169545) at a dilution of 1:600. After six washes with TBS-T to remove unbound primary antibodies, the membranes were incubated with HRP-conjugated secondary antibodies for 1 h at room temperature on a shaker. Goat anti-rabbit IgG-HRP (ab6112, Abcam, Cambridge, UK) was used as the secondary antibody at a dilution of 1:1000 for mouse sENG, while a dilution of 1:1500 was used for human sENG. Following six additional washes with TBS-T, protein bands were visualized using the ChemiDoc™ MP Imaging System (Bio-Rad Laboratories, CA, USA). Band intensities were quantified using Image Lab software (version 6.1, Bio-Rad Laboratories). To ensure equal protein loading, signals were first normalized to the total protein level visualized by Ponceau S staining. The normalized values were then expressed relative to the control group, which was set to 100% to allow for comparative analysis. Potential non-specific interference was evaluated by omitting the primary antibody and by normalizing to total protein loading, ensuring reliable and specific detection of sENG.

The selection of these antibodies and their optimized dilutions was based on preliminary experiments and manufacturer recommendations, and our previous work with these antibodies^7^, ensuring sensitive and specific detection of the target proteins across different sample types. To detect sENG in mouse plasma and cell culture media, we performed Western blotting using a primary antibody that typically recognizes a membrane-bound ENG at approximately 90–100 kDa. However, as ENG can undergo proteolytic cleavage, the soluble form can appear at lower molecular weights^28–30^. In our analysis, sENG from mouse samples was detected as bands ranging from ~ 46 to 80 kDa, while human sENG appeared between ~ 46 and 100 kDa. These variations are likely due to cleavage at different sites on the membrane-band ENG protein. Our experimental findings support this interpretation.

This extensively refined and detailed high-sensitivity protocol elaborates on our previously established method^7^.

### Statistical analysis

Data were analysed using GraphPad Prism software (version 10, GraphPad Software Inc., San Diego, CA, USA). Relative sENG levels were expressed as median with interquartile range. Group comparisons were performed using the non-parametric Mann–Whitney test. *P*-values < 0.05 were considered statistically significant.

## Results

Concentrated plasma samples from all groups, both in vivo and in *vitro*, demonstrated sufficient protein content, as confirmed by the Pierce™ BCA Protein Assay Kit. Western blot analysis successfully detected mouse sENG and human sENG across all sample types. The optimized antibody dilutions ensured proper detection of these proteins, with clear band visualization achieved using the ChemiDoc™ MP Imaging System.

### Relative quantification of mouse sENG in plasma

**Fig. 1:**
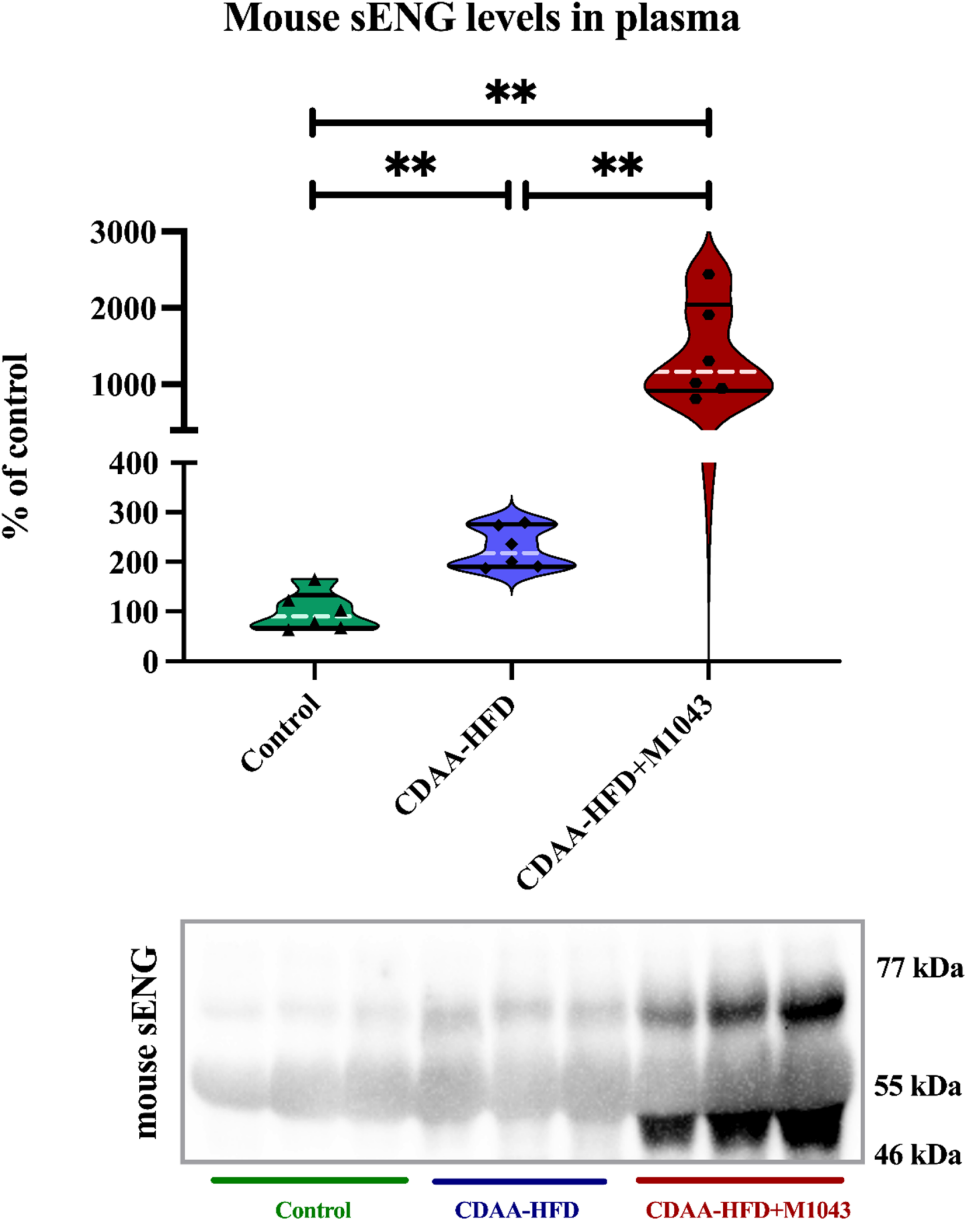
Effect of M1043 on sENG levels in plasma. Data are presented as median with interquartile range. Mann–Whitney test, **p < 0.01; 6 animals per group. Two gels were run simultaneously and detected together using the same ChemiDoc™ MP Imaging System and settings. Each gel contained 3 samples from the control group, 3 samples from the CDAA-HFD group, and 3 samples from the CDAA-HFD+M1043 group. Both gels were processed in parallel under identical conditions, including electrophoresis, protein transfer, blocking, antibody incubation, chemiluminescent detection, and exposure. Automatic exposure settings were applied consistently across all blots, ensuring uniform exposure times. Blots were cropped solely to fit the figure layout; no lanes or samples were removed or rearranged. Full, uncropped blots, along with Ponceau S staining, are provided in Figs. S3 and S7. Image processing, including brightness and contrast adjustments, was performed uniformly across entire images using Image Lab software. This approach ensures transparency and complies with the journal’s image integrity policy. Specificity of the secondary antibody was confirmed by omission of the primary antibody, which resulted in no detectable signal under identical exposure conditions (see Fig. S5).

Western blot quantification revealed significant differences in mouse sENG levels among the experimental groups. Compared to the control group, the MASH-induced group exhibited a marked increase in sENG levels, consistent with the progression of metabolic MASH. Notably, in the rescue group treated with M1043, plasma levels of sENG were significantly elevated compared to both the control group and the MASH mice. This upregulation of sENG suggests that M1043 treatment may induce shedding of membrane-bound ENG, leading to its release into the bloodstream. This hypothesis is supported by the detection of distinct bands corresponding to sENG in the CDAA-HFD+M1043 group (Fig. 1).

To demonstrate the reliability of the Western blot-based method, we also measured sENG levels by ELISA, which was possible in the control and CDAA-HFD diet groups without interfering mAb treatment. The results were comparable to those obtained in the initial experimental groups, suggesting that the Western blot-based method provides consistent and interpretable results. The supporting data are included in Fig. S1.

### Relative quantification of human sENG in culture media

**Fig. 2:**
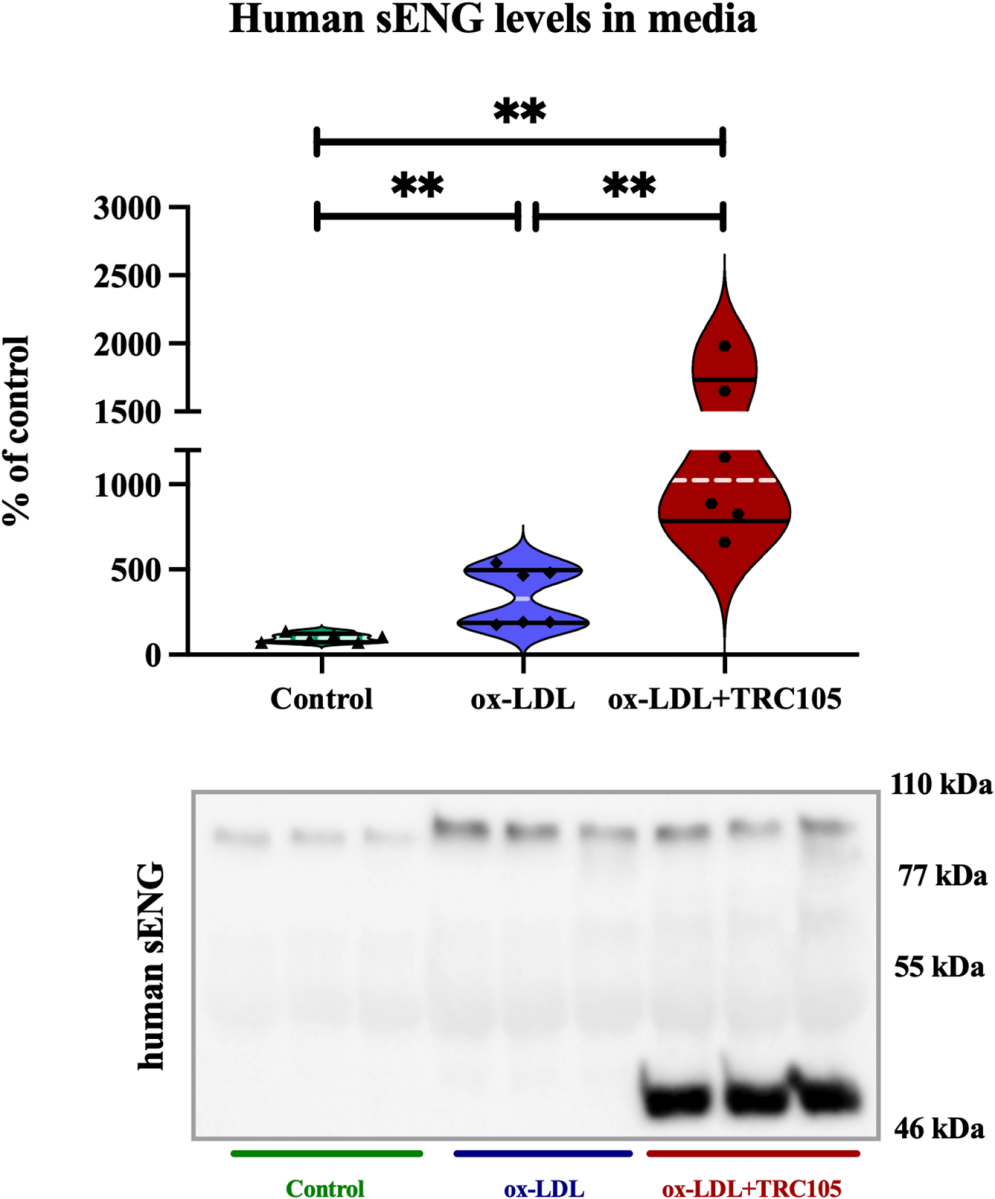
Impact of TRC105 on human sENG levels in media. Data are presented as median with interquartile range. Mann–Whitney test, **p < 0.01; (n = 6), showing representative figures from 3 independent experiments. Two gels were run simultaneously and detected together using the same ChemiDoc™ MP Imaging System and settings. Each gel contained 3 samples from the control group, 3 samples from the ox-LDL group, and 3 samples from the ox-LDL+TRC105 group. Both gels were processed in parallel under identical conditions, including electrophoresis, protein transfer, blocking, antibody incubation, chemiluminescent detection, and exposure. Automatic exposure settings were applied consistently across all blots, ensuring uniform exposure times. Blots were cropped solely to fit the figure layout; no lanes or samples were removed or rearranged. Full, uncropped blots, along with Ponceau S staining, are provided in Figs. S4 and S8. Image processing, including brightness and contrast adjustments, was performed uniformly across entire images using Image Lab software. This approach ensures transparency and complies with the journal’s image integrity policy. Specificity of the secondary antibody was confirmed by omission of the primary antibody, which resulted in no detectable signal under identical exposure conditions (see Fig. S6).

In vitro analysis of culture media from HHSECs revealed changes in sENG expression following treatment with ox-LDL and TRC105. Treatment with ox-LDL significantly increased sENG levels compared to untreated controls. Pre-treatment with TRC105 further upregulated sENG levels in the ox-LDL-induced group, demonstrating TRC105’s ability to enhance the shedding of ENG (Fig. 2). Similarly to the previous mouse model, in human samples, TRC105-induced shedding in the ox-LDL + TRC105 group produced several ENG bands (~ 50–100 kDa), consistent with the soluble form of ENG.

To assess the reliability of our Western blot–based method, sENG levels were additionally measured by ELISA, which was possible in the control and ox-LDL-induced groups without interfering mAb treatment. The observed results closely matched those from the primary experimental groups, indicating that the Western blot-based method yields consistent and reproducible data. Supporting results are provided in Fig. S2.

## Discussion

The primary objective of this study was to verify a cost-effective and specific Western blot-based method for the accurate quantification of sENG in mouse plasma during a preclinical study evaluating the therapeutic mouse anti-ENG mAb M1043 and human anti-ENG TRC105. In this study, we provide an updated and more detailed experimental protocol details compared to our previous research paper^7^, showing the modifications in several methodological aspects. For instance, we included full details on sample preparation, such as centrifugation speed/time, temperatures, use of the BCA protein assay, antibody dilution ratios, species-specific application, and cleavage-related molecular weight interpretation, explaining molecular weight variations reflecting proteolytic cleavage of membrane-bound ENG.

By overcoming the critical limitations of conventional methods, such as ELISA, which is prone to epitope blocking with therapeutic mAbs, and the high costs of advanced techniques like LC–MS/MS, this protocol addresses a significant gap in preclinical research. Comparative analysis in biological systems is essential for assessing therapeutic mAbs, given the central role of these systems in angiogenesis, inflammation, and fibrosis. Our findings demonstrate that this method not only mitigates the shortcomings of existing techniques but also establishes a practical, reproducible, and accessible solution for soluble biomarker analysis in both preclinical models and clinical studies treated with therapeutic antibodies, as previously established in our earlier investigation^7^. This innovative approach paves the way for enhanced biomarker evaluation in translational research.

A key challenge in quantifying sENG lies in the limitations of ELISA, which has long been considered the gold standard due to its high sensitivity and throughput^11–14^. However, ELISA’s reliance on antibody-antigen interactions renders it highly susceptible to interference when used in models treated with therapeutic mAbs. Indeed, Liu et al. aimed to evaluate sENG levels after TRC105 treatment in patients with advanced cancer to evaluate potential assay interference. TRC105 was initially tested using both the R&D Quantikine CD105 Immunoassay and the Aushon CD105 SearchLight Immunoassay kits. In the R&D assay, sENG levels decreased by approximately 20% at a molar ratio of sENG to TRC105 of 1:100 and were completely undetectable at ratios ≥ 1:1000, indicating significant interference. In contrast, the Aushon CD105 SearchLight Immunoassay kits demonstrated minimal interference, maintaining accurate sENG detection even at a molar excess of TRC105 as high as 1:10,000. Unfortunately, the Aushon CD105 SearchLight Immunoassay kit is not available anymore. Interestingly, Sumner et al. demonstrated a similar issue, showing that the Human Quantikine vascular endothelial growth factor (VEGF) ELISA kit is unable to accurately measure VEGF in the presence of anti-VEGF drugs^31^. This underscores a major limitation of ELISA in such contexts. The inability of ELISA to provide reliable results in these scenarios highlights the urgent need for alternative methods.

**Fig. 3:**
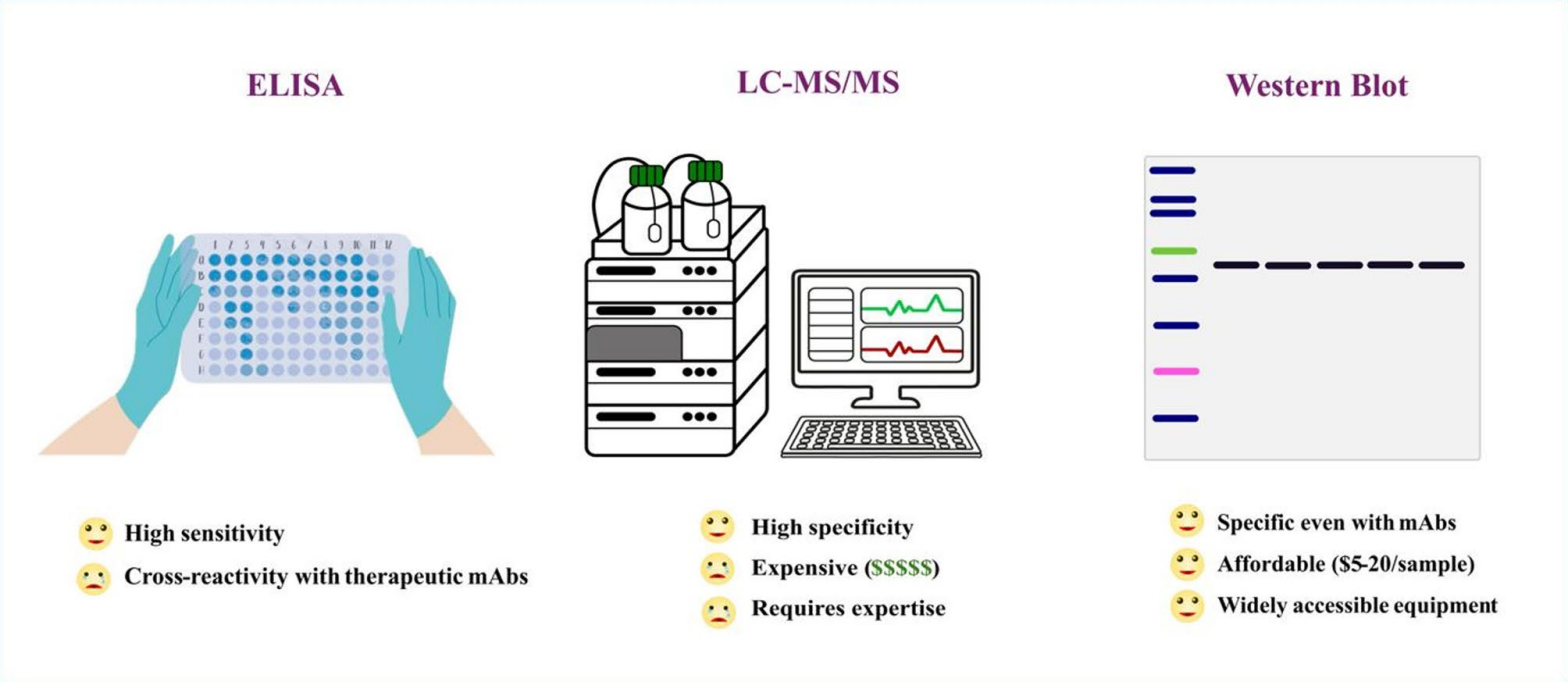
Overview of methods for quantifying soluble proteins after mAb treatment in biological samples. ELISA, while sensitive and high throughput, is prone to interference in samples treated with therapeutic mAbs, leading to unreliable results. Advanced techniques such as LC–MS/MS offer improved sensitivity and specificity but are limited by high costs, complex procedures, and specialized equipment requirements. In contrast, the Western blot–based protocol presented here provides a cost-effective and accessible alternative, balancing affordability with reliable data quality. The figure has been created in Inkscape (version 1.4).

Advanced techniques such as LC–MS/MS and proximity extension assays offer high sensitivity and specificity but remain inaccessible to most research laboratories due to their high costs, reliance on specialized expertise, and complex sample preparation requirements^24,32^. For instance, proximity extension assays depend on proprietary platforms (e.g., Olink) with reagent costs ranging from $50 to $200 per sample (USD), making them impractical for widespread use^24^. Similarly, LC–MS/MS requires expensive instrumentation, often costing $500,000 to $1,000,000 (USD), along with extensive training and maintenance^33–35^. In contrast, our Western blot-based protocol leverages widely accessible laboratory equipment and consumables, with costs as low as $5 to $20 per sample (USD)^36–38^. This affordability, combined with the method’s straightforward protocols, makes it an ideal solution for researchers seeking a balance between cost-effectiveness and robust data quality. Figure 3 summarizes ELISA limitations, LC–MS/MD challenges, and the advantages of our Western blot protocol.

**Fig. 4:**
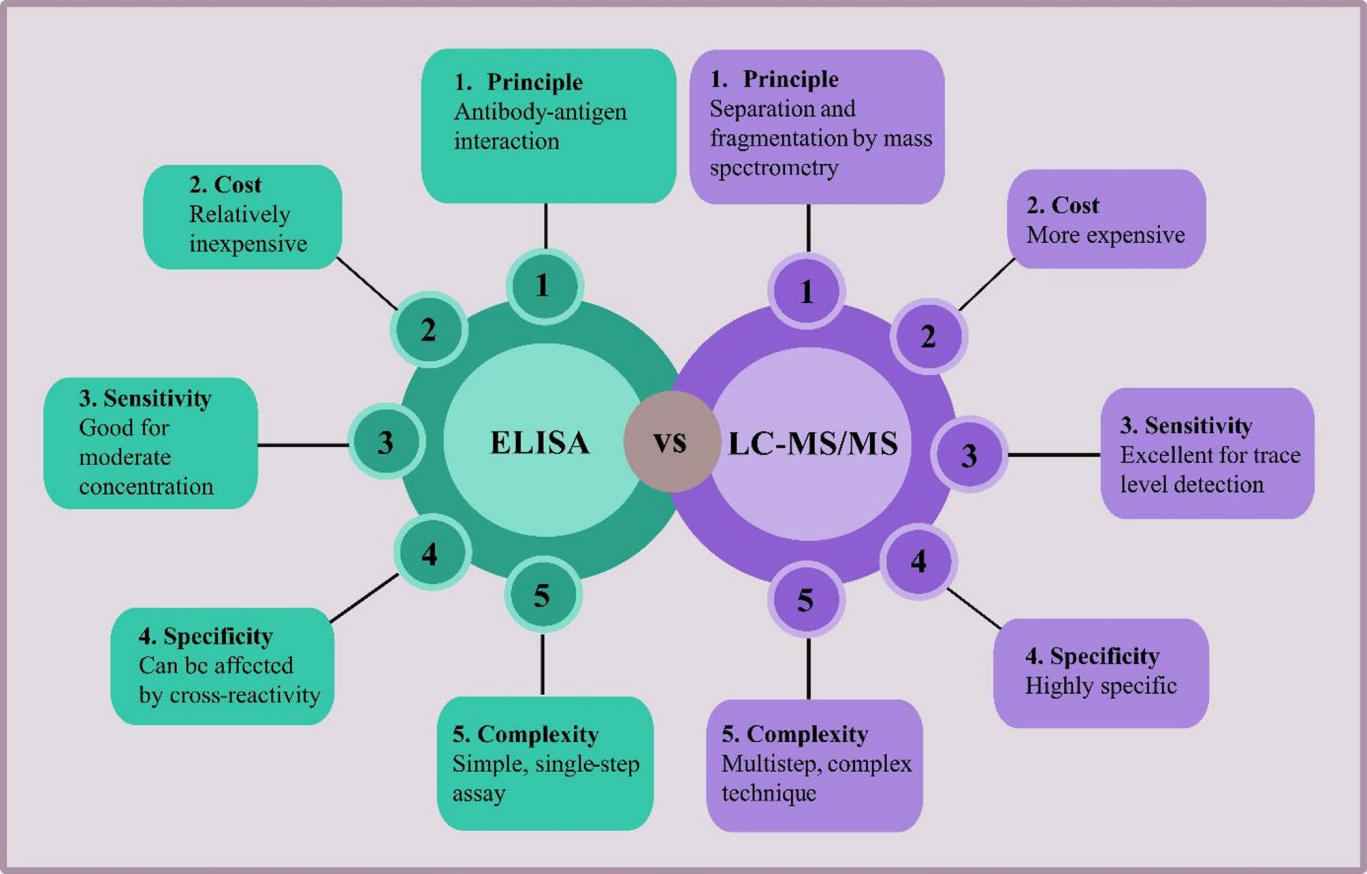
Comparative overview of ELISA and LC–MS. The figure summarizes key differences between ELISA and LC–MS in terms of analytical principle, detection limit, sensitivity, specificity, cost, and operational complexity. ELISA offers robust specificity, lower cost, and ease of use but has relatively higher detection limits, whereas LC–MS provides superior sensitivity and multiplexing capacity at the expense of higher cost and technical demands.

ELISA and LC–MS each offer distinct advantages for sENG detection. ELISA is cost-effective, simple, and highly specific but has higher detection limits, whereas LC–MS provides superior sensitivity and multiplexing capacity at greater cost and complexity. Figure 4 summarizes these differences in principle, cost, sensitivity, specificity, and complexity, highlighting ELISA’s suitability for routine use and LC–MS for highly sensitive or exploratory analyses.

By providing simultaneous insights into multiple soluble biomarkers that are the target of the treatment antibody, this approach enables researchers to evaluate the broader impact of treatments on disease mechanisms, offering a more comprehensive understanding of therapeutic efficacy. Moreover, the versatility of this method, demonstrated by its successful application to both mouse plasma and human cell culture media, makes it a valuable tool for translational research, effectively bridging preclinical and clinical investigations. Additionally, such an accessible method could facilitate the monitoring of treatment efficacy in patients receiving mAb therapies, offering a practical tool for guiding therapeutic decisions and tracking disease progression.

Although ELISA and LC–MS/MS dominate due to automation and precision in untreated samples, our Western blot-based method uniquely enables interference-free detection of sENG in mAb-treated contexts. By concentrating plasma and leveraging molecular weight separation, it overcomes limitations of low protein abundance, offering accessible, specific, and cost-effective detection and filling a critical methodological gap in the field.

Our results demonstrate that the optimized Western blot-based protocol is a reliable method for quantifying sENG in the presence of mAbs used for the treatment/blockage of ENG membrane ENG effects. Unlike ELISA, which is limited by epitope masking and inability to differentiate free from Ab-bound ENG, our approach remains robust under mAb treatment. While LC–MS/MS offers high sensitivity, it is not widely accessible and requires advanced instrumentation and technical expertise. By addressing these limitations, our protocol provides an accessible, reproducible, and practical tool for investigating sENG and related proteins in therapeutic contexts, offering a clear advantage over existing methods.

Despite its strengths, the Western blot method has limitations. Its semi-quantitative nature requires densitometry for comparisons, which introduces variability compared to the automation of ELISA^39,40^. While Western blotting is generally less precise than techniques such as ELISA or mass spectrometry for protein quantification, it remains a valuable tool for comparative analysis in biological systems. Despite its labour-intensive nature and the need for sample concentration, a factor that requires careful optimization to prevent protein degradation, the method offers significant advantages in terms of affordability, specificity, and reproducibility. These strengths are particularly relevant in studies involving therapeutic mAbs, where epitope blocking may limit the reliability of other assays. To enhance quantification accuracy, incorporating known concentrations of the target protein to generate a standard curve can enable more precise measurement by comparing sample signals to those of established standards. Future refinements will prioritize establishing calibration curves with recombinant sENG standards to enable absolute quantification, thereby enhancing the robustness of the current semi-quantitative framework.

Future directions include automating Western blot workflows using capillary-based systems (e.g., ProteinSimple Jess) to enhance throughput and reduce manual labor^41–43^. Integrating this method with absolute quantification techniques, such as targeted mass spectrometry, could further improve precision while maintaining cost-effectiveness. Furthermore, adapting this protocol for other soluble biomarkers facing similar epitope blocking challenges, such as VEGF or TGF-β, could expand its applicability across diverse disease contexts.

This method’s broad applicability, enabling detection of low-abundance, Ab-bound soluble biomarkers, supports both preclinical research (e.g., evaluating mAb effects across multiple disease models) and clinical practice (e.g., monitoring therapeutic response or disease progression in patients receiving mAb therapies). Its accessibility, reproducibility, and interference-free detection underscore its potential to bridge preclinical findings and translational applications, advancing cardiometabolic, vascular, and oncology research.

## Conclusion

In summary, this study presents a verified, cost-effective, and accessible Western blot-based protocol for the semi-quantitative evaluation of soluble proteins in models treated with therapeutic mAb. Using sENG as the primary target, we demonstrated the method’s specificity and robustness in both in vivo (mouse plasma, with the murine mAb M1043) and in vitro (human cell culture media, with the human-specific mAb TRC105) settings. By overcoming key limitations of ELISA epitope blocking and the high costs of techniques such as LC–MS/MS, this protocol offers a practical and reproducible solution for soluble biomarker analysis. Its versatility and affordability make it a valuable tool for both preclinical and clinical studies, facilitating therapeutic monitoring and the development of targeted treatments.

## Supplementary Information

Below is the link to the electronic supplementary material.

Supplementary Material 1

## Data Availability

The original contributions of this study are detailed in the article; further inquiries can be directed to the corresponding author.
