# Supplementary material for: Cost-effective method for semi-quantitative analysis of soluble endoglin in biological samples after anti-endoglin monoclonal antibody treatment
Source: Sci Rep. 2025 Oct 30;15:38066. doi: 10.1038/s41598-025-21972-w (PMC12575752; doi:10.1038/s41598-025-21972-w)

Supplementary Data

Measurement of sENG via ELISA

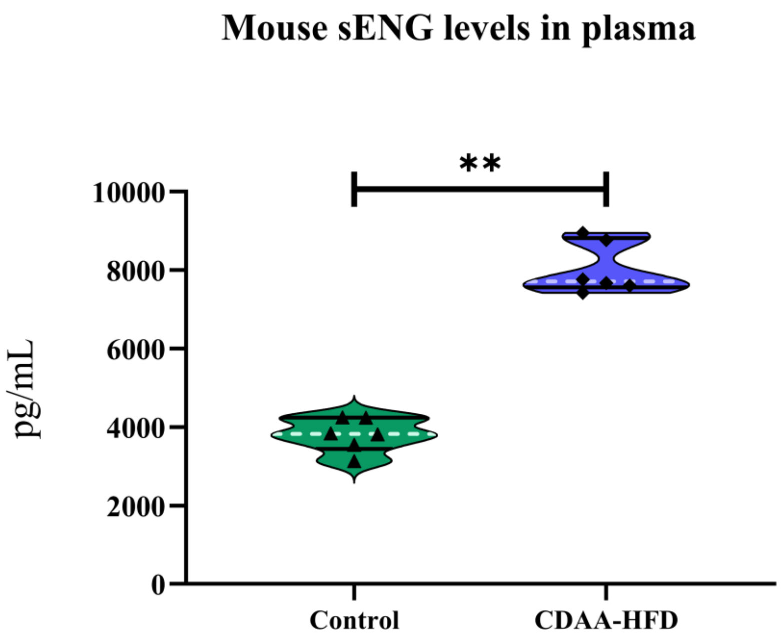

Figure S1: sENG levels in independent control and CDAA-HFD groups without monoclonal antibody treatment. Data are presented as median with interquartile range. Mann-Whitney test, ** $p < 0.01$; 6 animals per group.

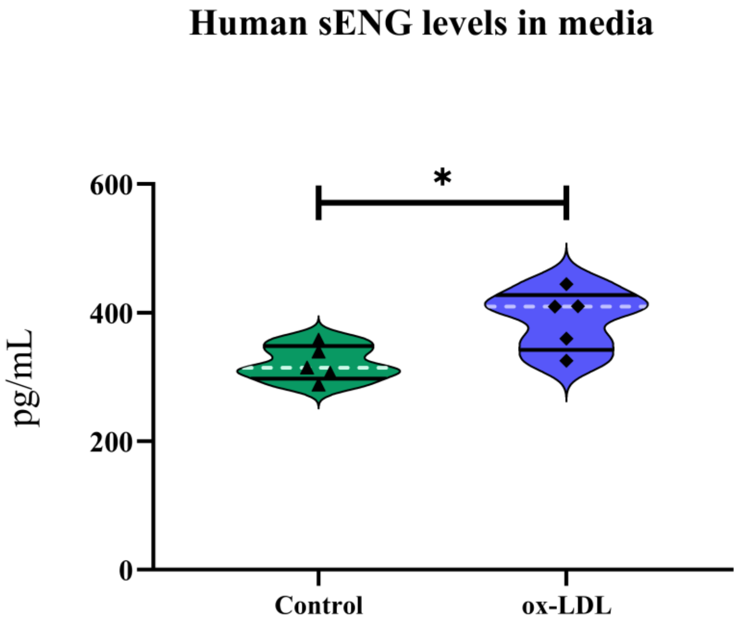

Figure S2: sENG levels in independent control and ox-LDL induced groups without monoclonal antibody treatment.. Data are presented as median with interquartile range. Mann-Whitney test, *$p < 0.05$; n = 5, showing representative figures from 3 independent experiments.

Supplementary Data

Figure S3: Mouse sENG

Gel 1

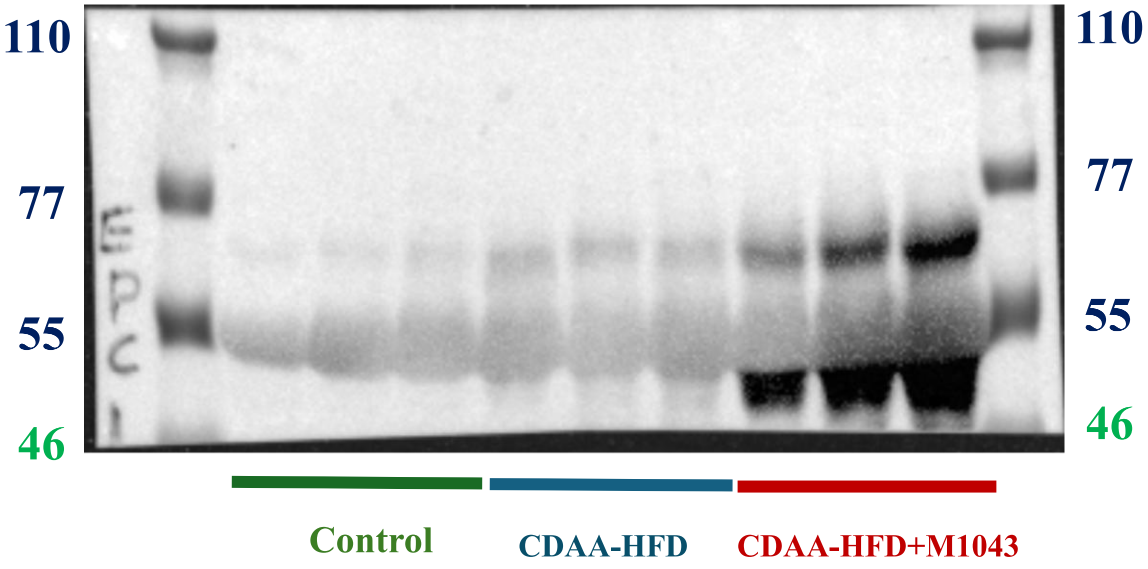

Gel 2

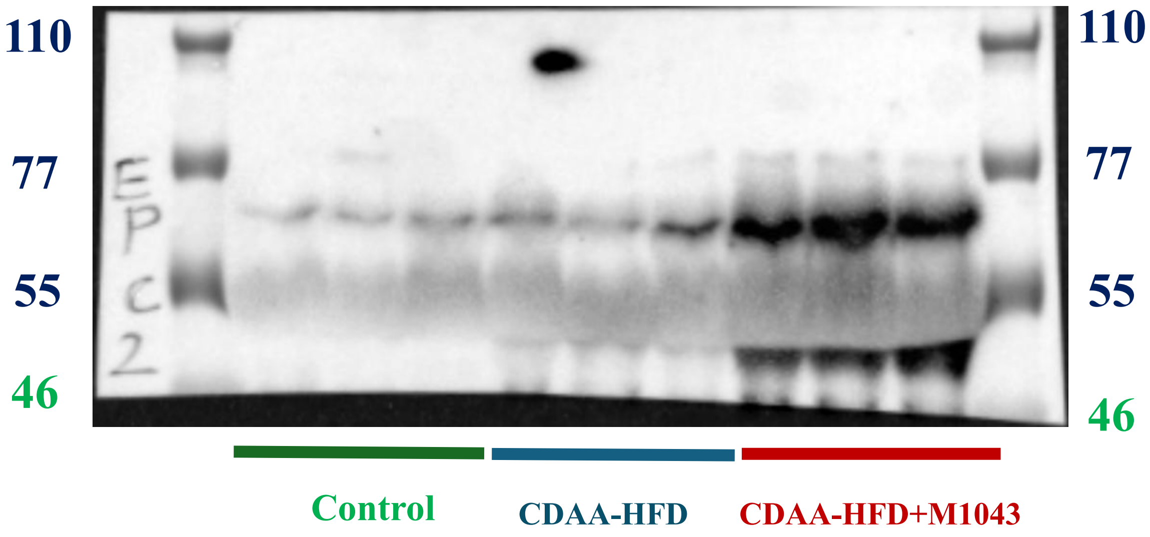

Supplementary Data

Figure S4: Human sENG

Gel 1

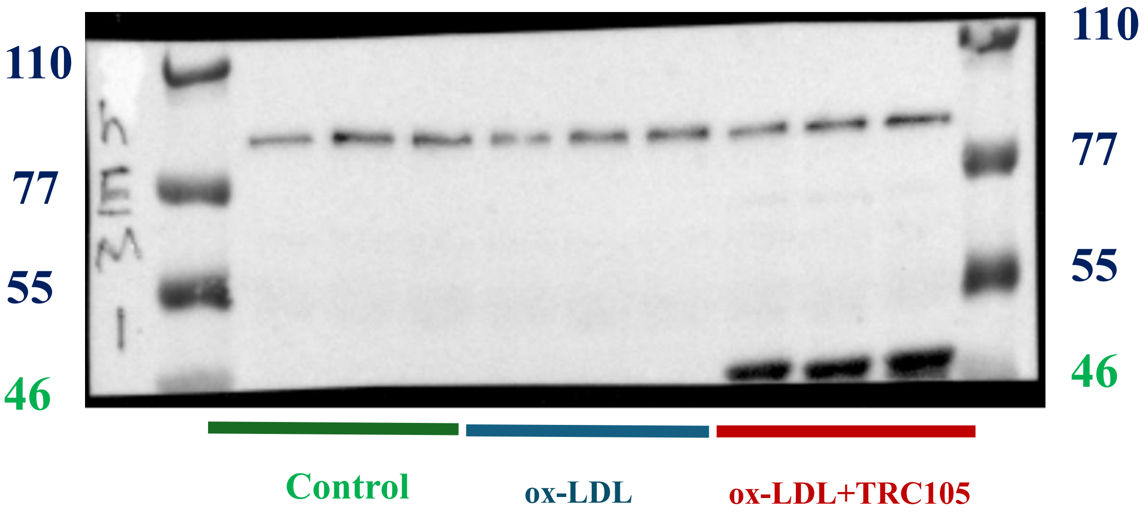

Gel 2

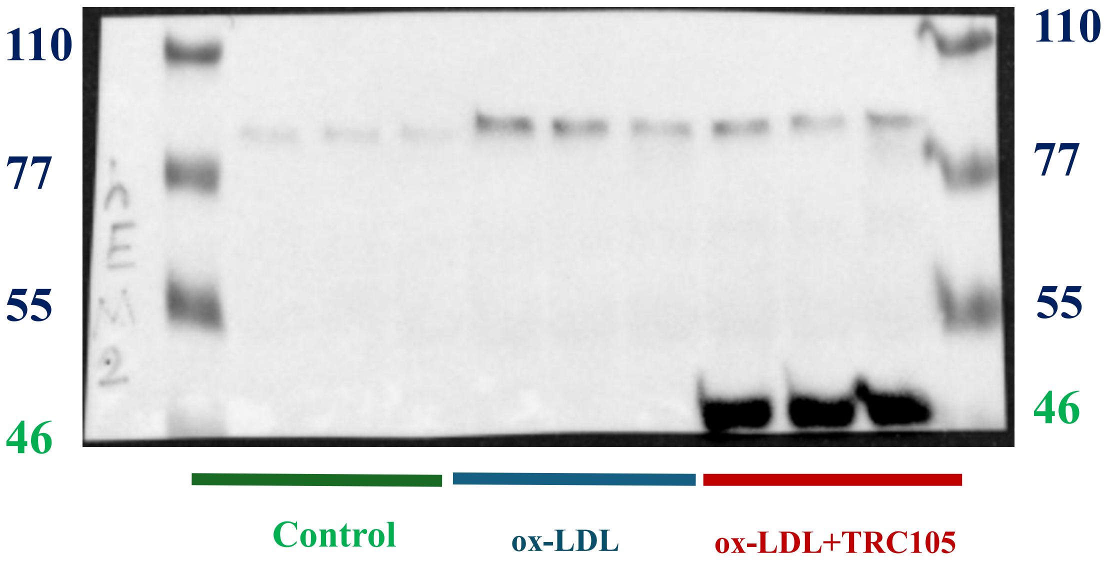

Supplementary Data

Figure S5: Confirmation of secondary antibody specificity by omission of primary antibody (mouse ENG)

Gel 1

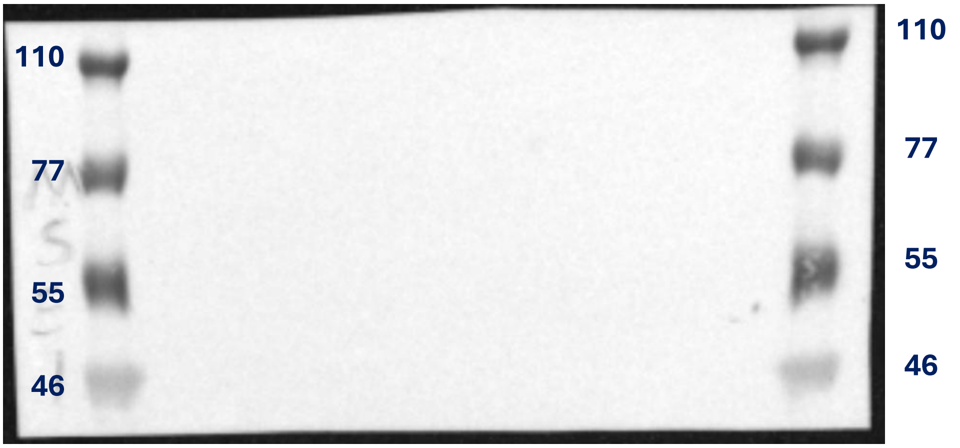

Gel 2

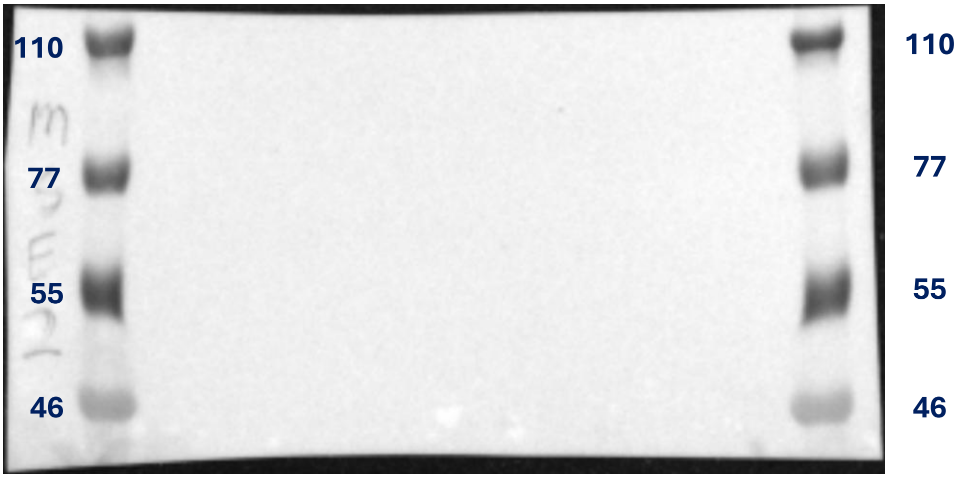

Supplementary Data

Figure S6: Confirmation of secondary antibody specificity by omission of primary antibody (human ENG)

Gel 1

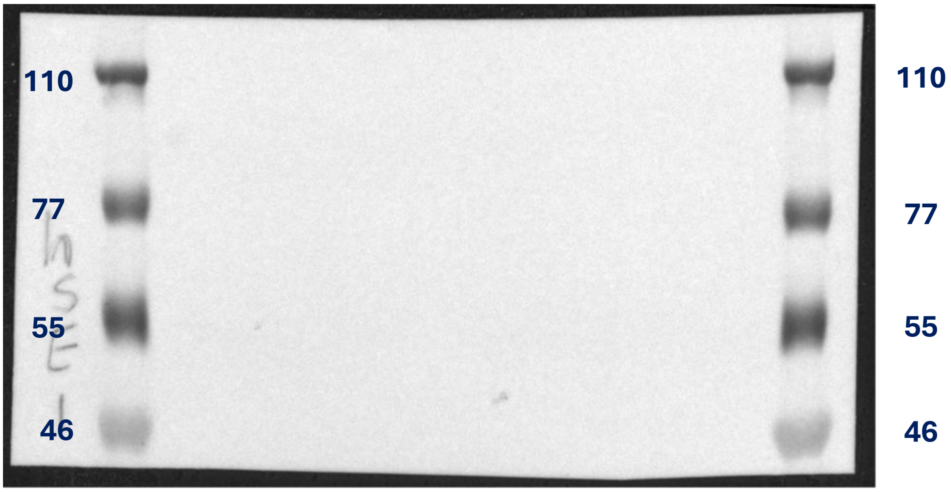

Gel 2

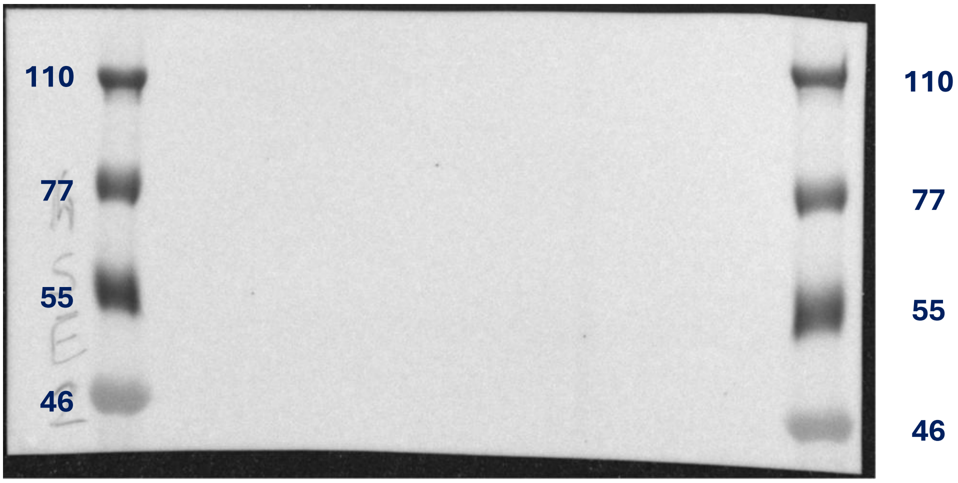

Supplementary Data

Figure S7: Ponceau S staining of mouse plasma samples

Control

CDAA-HFD

CDAA-HFD+M1043

Gel 1

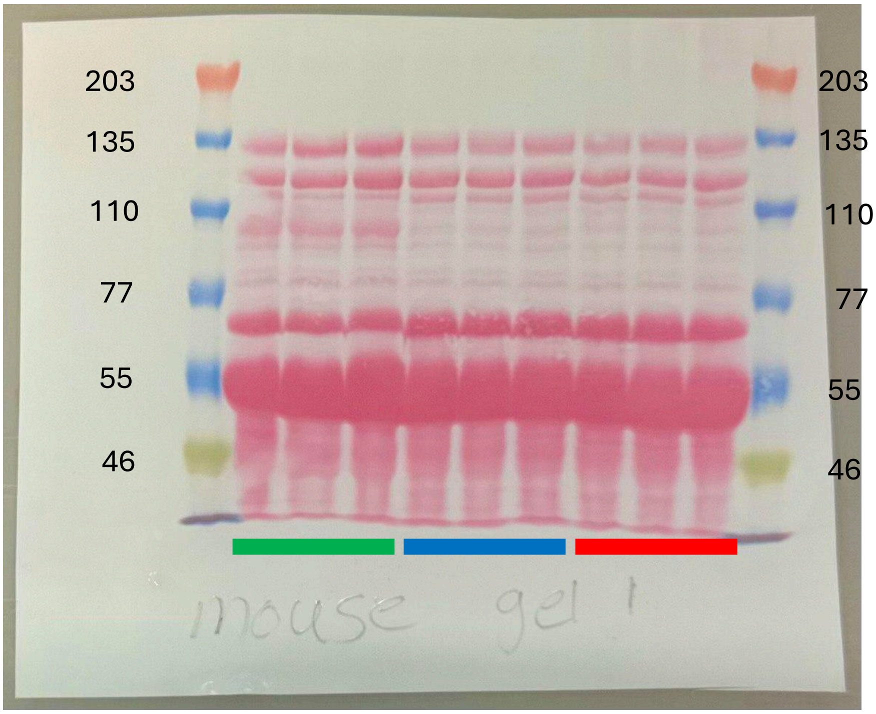

Gel 2

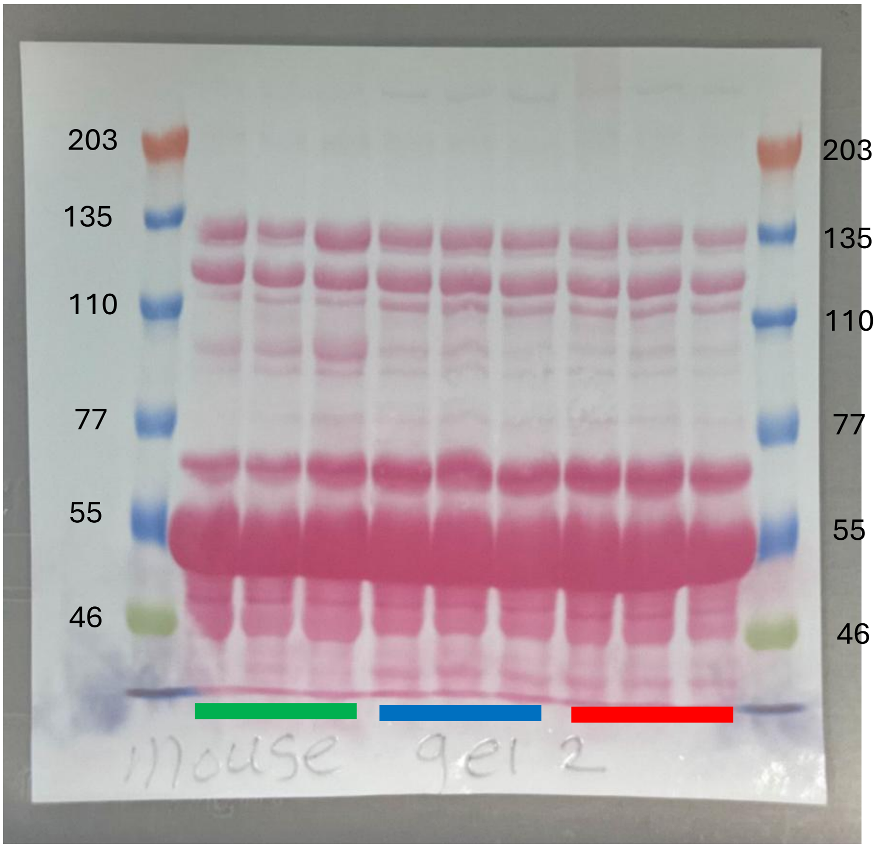

Supplementary Data

Figure S8: Ponceau S staining of human culture media samples

Control

ox-LDL

ox-LDL+TRC105

Gel 1

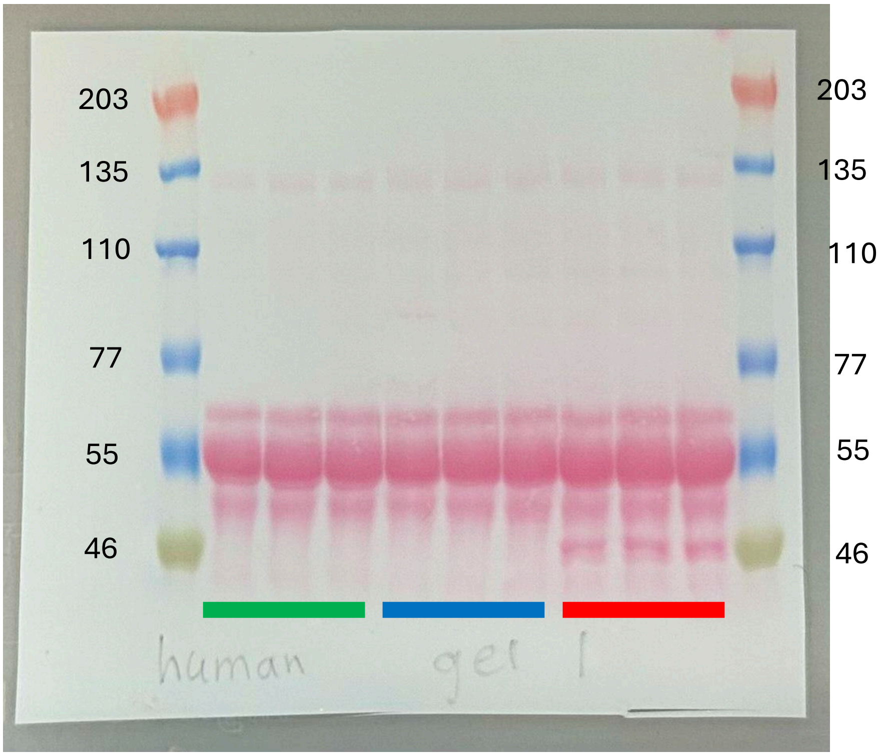

Gel 2

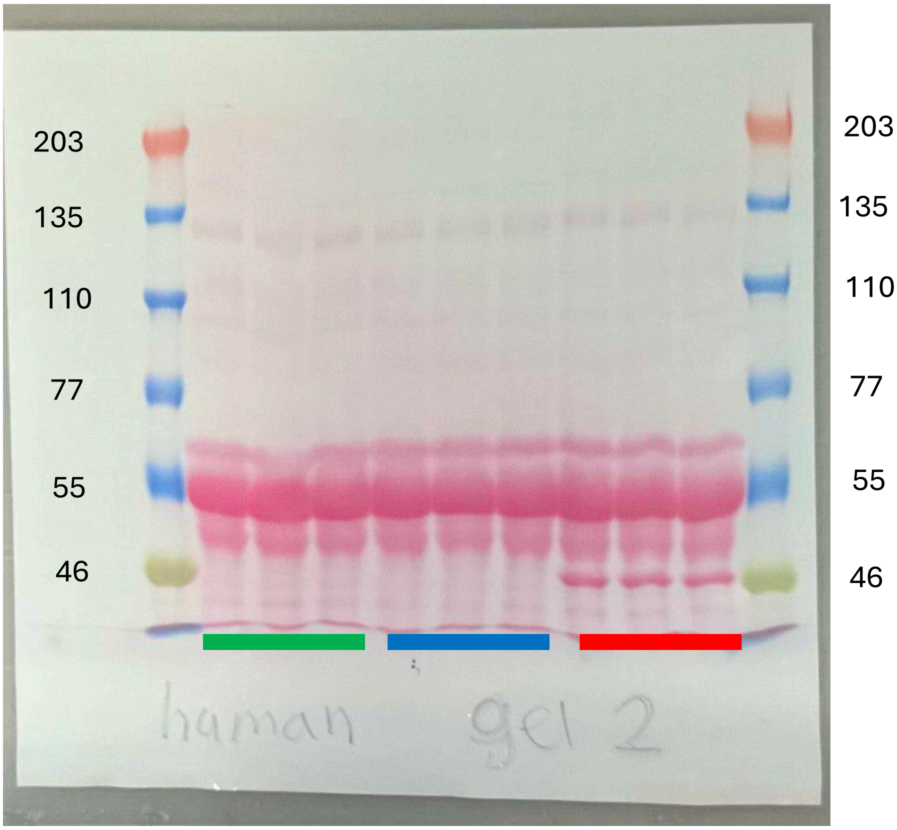

Supplement: Supplementary file 1 — Supplementary Material 1 [file 41598_2025_21972_MOESM1_ESM.pdf]
